# Primary cervical malignant melanoma that was successfully treated with pembrolizumab: a case report and literature review

**DOI:** 10.3389/fonc.2024.1400257

**Published:** 2024-06-26

**Authors:** Mengpei Zhang, Rutie Yin, Liang Song, Lan Zhong, Ruiyu Wang

**Affiliations:** ^1^ Department of Obstetrics and Gynecology, West China Second University Hospital of Sichuan University, Chengdu, China; ^2^ Key Laboratory of Birth Defects and Related Diseases of Women and Children (Sichuan University), Ministry of Education, Chengdu, China

**Keywords:** primary cervical malignant melanoma, pembrolizumab, case report, cervical cancer, immunotherapy

## Abstract

Primary malignant melanoma (MM) arising from the cervix is an exceedingly rare occurrence, and patients diagnosed with this condition often face a dismal clinical prognosis. Here, we present a case study of a postmenopausal woman presenting with vaginal bleeding and a conspicuous 5-centimeter black mass on the cervix. Based on the staging criteria established by the International Federation of Gynecology and Obstetrics, she was diagnosed with stage IIB primary cervical MM. The patient underwent neoadjuvant therapy prior to a radical hysterectomy and a bilateral salpingo-oophorectomy. Subsequently, she completed 18 cycles of pembrolizumab therapy, achieving clinical complete remission. Notably, at the 31-month follow-up, there were no signs of recurrence. This successful treatment outcome serves as a valuable clinical reference for the management of primary cervical MM.

## Introduction

The National Cancer Database (NCDB), spanning the years from 1985 to 1994, revealed that cutaneous melanoma comprises 91.2% of all melanocytomas, with ocular melanoma accounting for 5.2%, mucosal melanoma accounting for 1.3%, and melanomas of unknown origin accounting for 2.2% (1). Cervical melanoma is classified as a mucosal melanoma and accounts for 3%–9% of all mucosal melanomas (2). Cervical melanoma carries a poor prognosis, and there remains no standardized and effective treatment approach. Despite significant advancements in the use of immune checkpoint inhibitors for the treatment of melanoma since 2015, a standardized protocol for managing cervical melanoma remains elusive, with particularly scant data on the application of immune checkpoint inhibitors in this context (3). Herein, we present a case of a patient diagnosed with stage IIB cervical melanoma. This individual underwent neoadjuvant therapy, followed by a radical hysterectomy and a bilateral salpingo-oophorectomy. Subsequently, the patient completed 18 cycles of pembrolizumab therapy, achieving clinical complete remission. Notably, at the 31-month follow-up, there were no signs of recurrence, providing valuable insights into the potential of immunotherapy in the management of cervical melanoma.

## Case report

**Figure 1:**
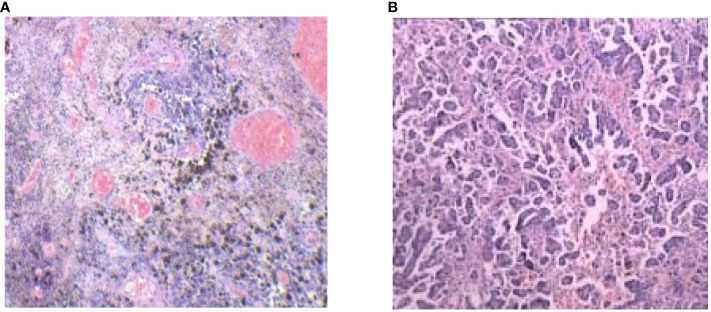
Pathologic examination of tumor tissue confirmed typical cervical MM histological features (hematoxylin and eosin stain 100/400).

A 77-year-old postmenopausal woman (para 2) presented to our hospital with a 2-month history of vaginal bleeding. Notably, she had a past history of tuberculosis, which was successfully treated with regular anti-tuberculosis therapy. Additionally, she was diagnosed with hypertension, coronary heart disease, and hypothyroidism over 10 years ago and is currently maintaining a stable condition with regular medication. A comprehensive physical examination revealed no abnormalities, and thorough dermatological, mucosal, and ocular inspections failed to detect any signs of primary melanoma. However, a significant finding was a 5 cm × 4 cm cauliflower-shaped dark brown lesion with a blood clot adhering to the surface of the cervix. Furthermore, a black nodule approximately 1 cm in diameter was observed at the 7 o’clock position on the posterior vaginal wall, located 2 cm from the fornix. Scattered melanin deposits were also visible on the lateral wall of the vagina. Gynecological examination suggested bilateral parametrial tissue invasion, although it did not extend to the lateral pelvic wall. A cervical biopsy revealed a round-cell tumor exhibiting solid growth and abnormal mitotic activity. The tumor cells were round-shaped and atypical, with conspicuous nucleoli indicative of malignant melanoma. Immunohistochemical results strongly suggest a diagnosis of malignant melanoma, based on positivity for HMB45, Melan-A, and S-100 and negativity for epithelial markers including P-CK, EMA, P16, P63, CK5/6, and P40. Vim positivity indicates a possible mesenchymal origin, while a high Ki67 positive rate (80%) suggests a tumor with high proliferative activity and potential for metastasis (**Figure 1**). However, a comprehensive evaluation, including clinical, histopathological, and radiological data, is necessary for a final diagnosis.

**Figure 2:**
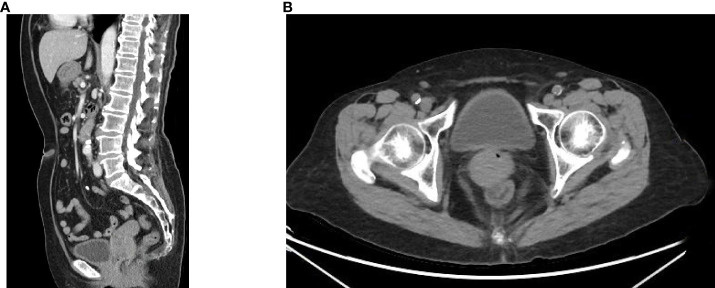
The CT scan before treatment revealed an enlarged cervix with inhomogeneous enhancement.

**Figure 3:**
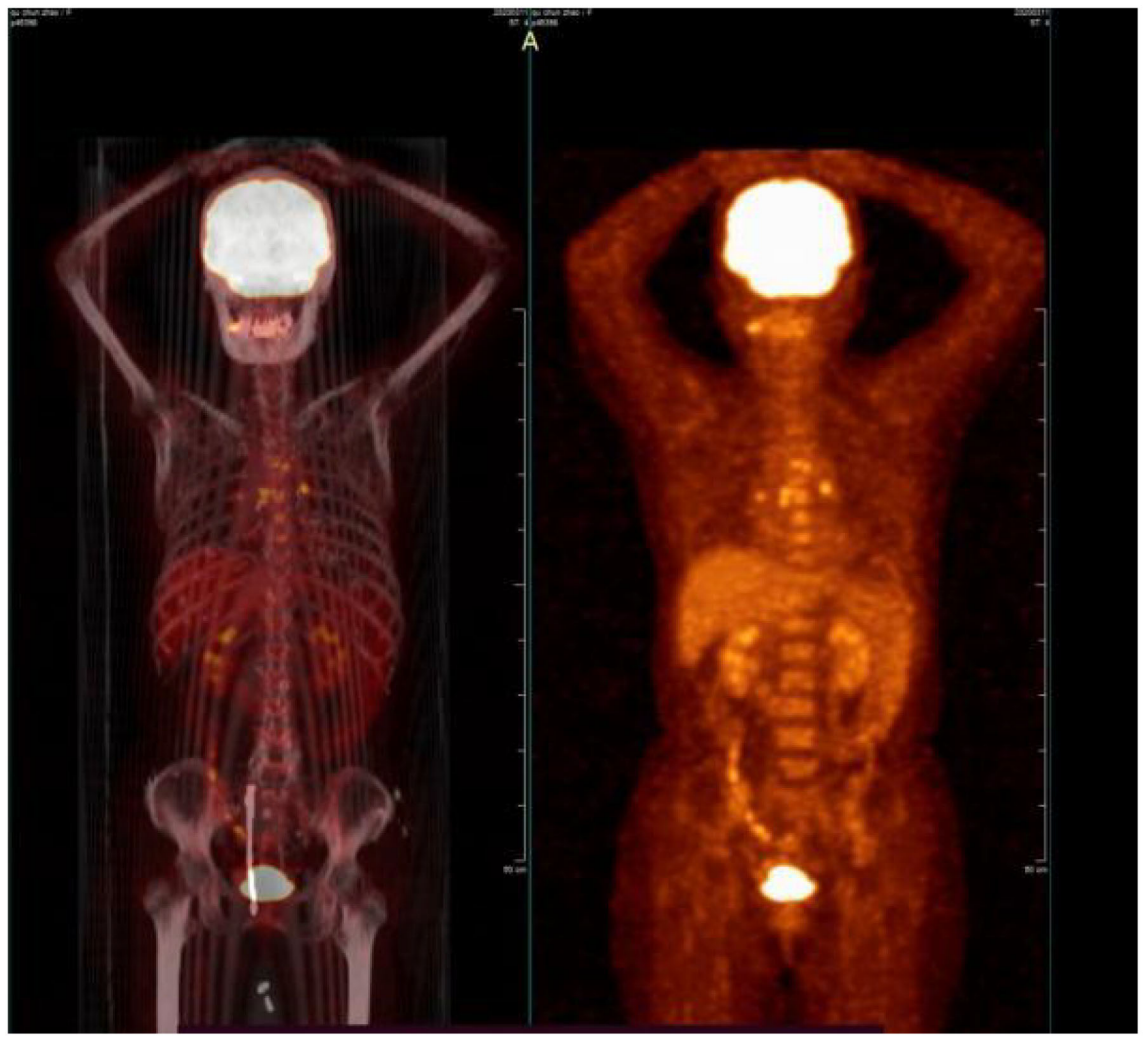
PET-CT before treatment showed that the mass was located in the cervix.

The pelvic CT scan indicates a relatively enlarged cervix with inhomogeneous enhancement. (**Figure 2**). Positron-emission tomography-computed tomography (PET-CT) also revealed increased fluorine-18-deoxyglucose intake lesions in the cervix without lymph node enlargement or any metastasis involving the skin or other mucosal sites (**Figure 3**). The patient was clinically diagnosed as stage IIB according to the International Federation of Gynecology and Obstetrics (FIGO 2018) classification system.

**Figure 4:**
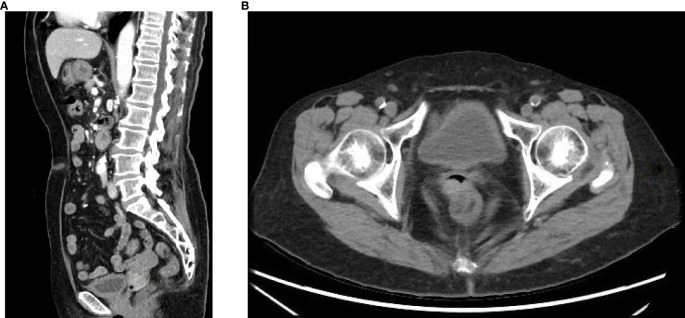
The pelvic CT scan showed that the cervical lesion had shrunk after neoadjuvant therapy.

**Figure 5:**
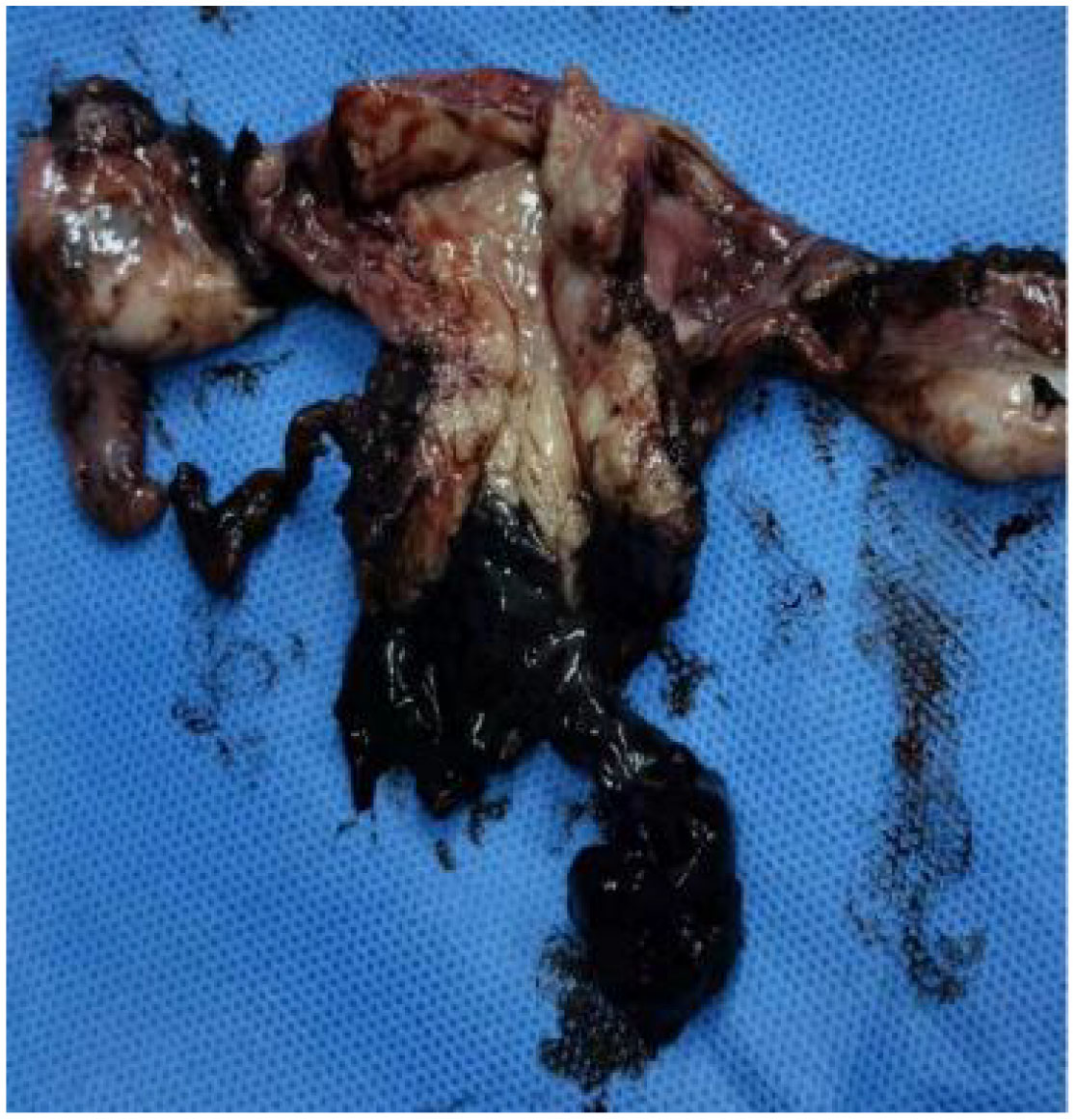
Surgical specimen.

**Figure 6:**
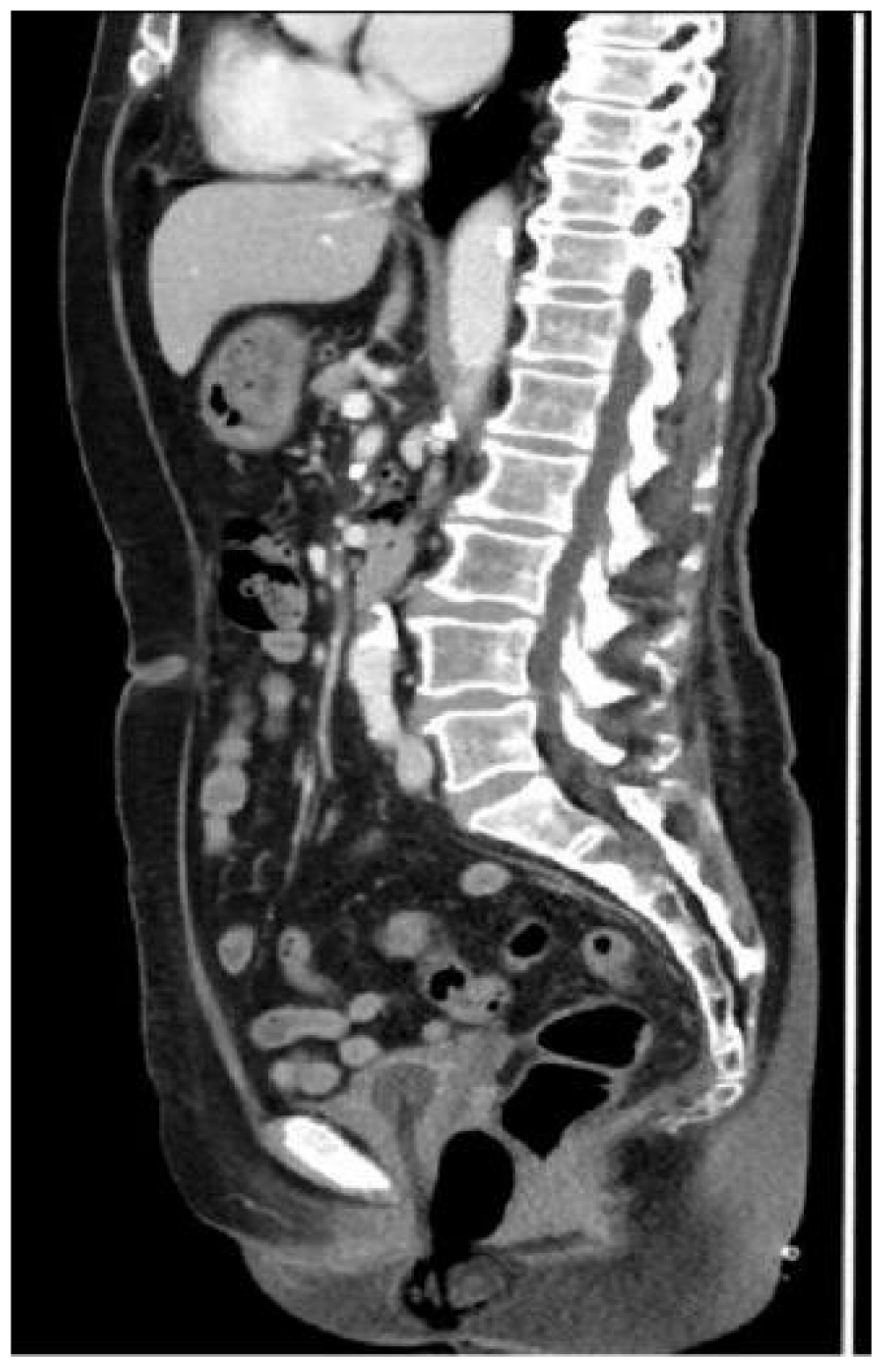
The pelvic CT scan showed that after the end of treatment, the patient achieved clinical complete remission.

Research indicates that dacarbazine demonstrates significant therapeutic effects in 15% to 20% of patients with mucosal melanoma. The combination of temozolomide and cisplatin chemotherapy has been shown to prolong relapse-free survival. Additionally, the combination of dacarbazine, cisplatin, and vinca alkaloids has an effective rate of up to 32% (4). Based on the chemotherapy experience with mucosal melanoma, the recommended primary chemotherapy regimen for cervical melanoma involves monotherapy or combination therapy, primarily using dacarbazine or its oral analog, temozolomide. The patient first received two cycles of neoadjuvant chemotherapy: dimethyl triazemo imidazole, carboxamide 200 mg d1 to d5, and cisplatin 30 mg d1 to d3 for two cycles. After two cycles of chemotherapy, the patient was assessed for no significant reduction in the cervical lesion. The treatment regimen was then switched to pabrolizumab 200 mg for three cycles. After previous treatment, the patient’s cervical lesion shrank (**Figure 4**). The total diameter of the target lesion decreased by approximately 28%, and the patient achieved SD according to the RECIST guideline (version 1.1) (5). After evaluation by the surgical professor, it was pointed out that the patient had a chance for surgery. Therefore, a radical hysterectomy and a bilateral salpingo-oophorectomy were performed. The size of the tumor was 5 cm × 4 cm (**Figure 5**). The postoperative results revealed a highly cellular neoplasm that was composed of a round-shaped atypical tumor with a conspicuous nucleolus. The immunohistochemical staining of Melan-A, HMB-45, and s-100, S-100 was positive. The NARS, BRAF, and KIT mutations were negative. The tumor invaded the middle 1/3 of the cervical mesenchyme, and the vaginal surgical margin of the specimen was positive. The tumor did not extend to the cervical-uterine junction, surgical resection margins of the pelvic sidewall, parametrial tissues, lymph nodes, or adnexa. After the operation, she underwent intravenous administration of 200 mg of pembrolizumab, administered every 3 weeks for 18 courses. This patient was assessed after the end of treatment and achieved clinical complete remission (**Figure 6**). She was reviewed every 3 months after treatment, and to this day, no signs of tumor recurrence have been detected. Throughout the time of treatment and follow-up, this patient developed immune-related dermatitis, which improved with the administration of hormonal therapy. This patient has survived for 49 months since the initial treatment and is now generally living a good life.

## Discussion

MM primarily occurs on the skin surface, with less than 2% of cases occurring in the female genitalia. Among these cases of MM in the female reproductive tract, the vulva or vagina are particularly common sites (6). However, primary MM of the cervix is extremely rare, and its prognosis is quite poor. Over the years, the incidence rate of primary cervical MM has not shown significant changes. Research suggests that radiation exposure, the influence of estrogen, and immunosuppression during pregnancy may be potential risk factors for this condition (7). Due to the rarity of its manifestations, the scarcity of evidence for optimal treatment strategies, and the absence of follow-up data, accurate survival predictions remain elusive. Regardless of staging and treatment methods, the prognosis is typically poor, with 87.5% of patients reported in the literature succumbing to the disease within 36 months of diagnosis. Reported survival times range from 6 months to 14 years, averaging 22.9 months. Based on a comprehensive review of available literature, the 5-year survival rate for primary cervical melanoma stands at 25% for stage I, 14% for stage II, and 0% for both stages III and IV (8, 9). Currently, there are no clear guidelines for the treatment of primary cervical MM. Aiping et al. first conducted a summary analysis of 149 cases of primary cervical melanoma. They discovered that patients who underwent radical hysterectomy, those with non-metastatic lymph nodes, and those who received lymphadenectomy had significantly improved survival rates. Among patients who underwent radical hysterectomy-based surgery, those who did not receive additional treatments had higher 24-month survival rates, while for those who underwent total hysterectomy, adding other treatments might be beneficial for extending median survival rates (10). Therefore, clinicians primarily rely on surgical standards for cervical squamous cell carcinoma when treating patients with primary cervical malignant melanoma. Decision-making regarding adjuvant therapy before or after surgery often depends on clinical experience (11).

The pathogenesis of female genital melanoma is largely unknown and is similar to that of other mucosal melanomas. Similar to other mucosal melanomas, c-KIT, BRAF, and NRAS mutations are frequently detected in female genital melanomas. However, in different studies, the proportion of mutations in each gene varies considerably between lesions in different sites, such as the vulva, vagina, and cervix (12, 13). Hou et al. found that PD-1 and PD-L1 were expressed in 75% and 56% of vulvovaginal melanomas. However, these data are not clear for cervical malignant melanoma (14). The development of vulvar, vaginal, and cervical melanomas may involve different molecular pathways. Therefore, molecularly targeted therapy and immunotherapy in vulvar, vaginal, and cervical malignant melanoma should be studied categorically.

As primary cervical malignant melanoma is extremely rare, there is no standard, agreed-upon treatment. Surgery is the first choice for early, feasible melanoma of the cervix. For these patients, surgery usually entails radical hysterectomy, pelvic lymph node dissection, and partial vagotomy vaginectomy with free surgical margins of at least 2 cm, but total extra-fascial hysterectomy has been reported in the literature in some cases (15, 16). The utilization of chemotherapy alone for the treatment of cervical malignant melanoma is not advocated and is mostly used for neoadjuvant chemotherapy or adjuvant treatment after surgery (16). Dacarbazine is used most widely for advanced-stage or recurrent disease, and about 15%–20% of patients respond to this treatment (17). Adjuvant pelvic radiotherapy may be considered for patients at high risk of recurrence, such as those with positive surgical margins, positive parametrium, and positive lymph nodes. Malignant melanoma is not sensitive to radiation, and conventional radiation therapy is ineffective. For patients who are not suitable for radical surgery, external pelvic irradiation ± intracavitary irradiation may be a palliative treatment modality (16). Karasawa et al. treated 23 patients with gynecologic genital tract malignant melanoma, including 14 vaginal MM, 6 vulval MM, and 3 cervical MM, with gynecologic genital tract malignant melanoma with carbon ion radiotherapy (C-ion RT). The 3-year local control and overall survival rates were 49.9% and 53.0%, respectively. The results of this study suggest that C-ion RT may become a non-invasive treatment option for gynecological melanoma (18).

In recent years, immunotherapy has shown encouraging results with a significantly improved prognosis in the treatment of cutaneous malignant melanoma. However, immunotherapy with immune checkpoint inhibitors has been poorly studied in mucosal melanoma, especially in cervical melanoma, with poor long-term clinical outcomes. The vast majority of studies were case reports, and their findings were inconsistent. Indini A. et al. reported in a case series, including seven cases of mucosal MM, that the role of immunotherapy in metastatic melanoma of the lower genital tract showed a response rate of 28% and was proved to have a poor prognosis (19). Reiko Suzuki et al. reported a case with primary MM of the uterine cervix that had a poor response to pembrolizumab and had an OS of 6 months (20). The study reported by Mayuka Anko et al. seems encouraging. They described a recurrent cervical MM case, and a patient with vaginal MM was diagnosed as stage IV with multiple metastases. After initiating nivolumab therapy, both patients were clinically diagnosed as being in remission without tumor progression (21). Until now, only five cases of cervical melanoma patients treated with immune checkpoint inhibitors have been reported. The first four cases showed poor outcomes, but the last case reported tumor reduction. Schiavone B. S. et al. described a patient who received ipilimumab before surgery and pembrolizumab after recurrence but passed away 19 months later (22). Kim M.S. et al. and Reiko Suzuk et al. reported similar cases with pembrolizumab post-surgery, but both patients died within 8 months and 6 months (20, 23). Noguchi T. et al. reported a case where nivolumab was used after recurrence with a limited 13-month survival (15). However, Anko M. et al. provided hope, reporting a patient with a recurrent pelvic tumor who achieved complete tumor disappearance with nivolumab and has been progression-free for 17 months (21). Despite limited cases and mixed outcomes, Anko M. et al.’s findings offer promise for future research and improved treatment options for cervical melanoma patients.

According to literature reports, the median OS benefit from immunotherapy with PD-1 or PD-L1 inhibitors in patients with mucosal melanoma is significantly shorter compared to patients with cutaneous melanoma (18 months vs. 45 months, P=0.003), and significant efficacy is only observed in a subset of patients (24). Therefore, chemotherapy remains the preferred postoperative adjuvant therapy for operable mucosal melanoma patients, while immunotherapy has a relatively lower recommendation level. However, for locally advanced, recurrent, or metastatic mucosal melanoma, immunotherapy may potentially enhance disease remission rates. In the case we reported, although the patient showed insensitivity to the preoperative chemotherapy regimen, immunotherapy appeared to demonstrate effectiveness. Given that the patient had comprehensive commercial health insurance that could adequately cover the high medical costs, she decided to continue immunotherapy postoperatively after thorough communication with the medical team. The final treatment outcome confirmed the effectiveness of our therapeutic approach, and the patient achieved significant clinical benefits. The final outcome demonstrated that our treatment measures were effective. The patient has survived a total of 49 months since initial treatment and has had no tumor recurrence to date. During the course of treatment, the patient repeatedly developed immune-related dermatitis, which improved after hormone therapy. No other severe adverse reactions were observed. And the side effects were within manageable limits, which proved that the use of pembrolizumab may be effective as a pre- and post-surgical adjuvant immunotherapy. It has been reported in the study of Eggermont A et al. that regardless of PD-1 ligand expression level and BRAF mutation status, pembrolizumab has been found to be associated with longer progression-free survival and overall survival in melanoma (25). In this patient, the NARS, BRAF, and KIT mutation genes were negative. This patient could show different molecular features compared to cutaneous MM and other cervical MM, so the clinic outcome was different. Although our case suggests that pembrolizumab is effective in the adjuvant treatment of primary cervical malignant melanoma, more data and randomized trials on mucosal MM, including cervical MM, are necessary to stratify successful cases.

## Conclusion

In summary, cervical primary MM is a rare type of cervical cancer with limited research. Surgical resection is the recommended treatment for early-stage disease. There is no standard treatment for advanced cervical primary MM. Based on this case report, surgery combined with immunotherapy may be a suitable option for locally advanced cervical primary MM.

## Data availability statement

The raw data supporting the conclusions of this article will be made available by the authors, without undue reservation.

## Ethics statement

Written informed consent was obtained from the minor(s)’ legal guardian/next of kin for the publication of any potentially identifiable images or data included in this article.

## Author contributions

MZ: Writing – original draft, Formal Analysis, Data curation. RY: Writing – review & editing. LS: Writing – review & editing, Methodology. LZ: Writing – review & editing, Software. RW: Writing – review & editing.
